# Novel Technologies for Target Delivery of Therapeutics to the Placenta during Pregnancy: A Review

**DOI:** 10.3390/genes12081255

**Published:** 2021-08-17

**Authors:** Gerald J. Pepe, Eugene D. Albrecht

**Affiliations:** 1 Department of Physiological Sciences, Eastern Virginia Medical School, Norfolk, VA 23507, USA; pepegj@evms.edu; 2 Departments of Obstetrics/Gynecology/Reproductive Sciences and Physiology, University of Maryland School of Medicine, Baltimore, MD 21201, USA

**Keywords:** spiral artery remodeling, pregnancy diseases, placenta, preeclampsia, peptide-mediated gene targeting, trophoblast-targeted nanoparticles, intra-placental gene therapy, targeted placental VEGF gene therapy, nonhuman primate

## Abstract

Uterine spiral artery remodeling is essential for placental perfusion and fetal growth and, when impaired, results in placental ischemia and pregnancy complications, e.g., fetal growth restriction, preeclampsia, premature birth. Despite the high incidence of adverse pregnancies, current treatment options are limited. Accordingly, research has shifted to the development of gene therapy technologies that provide targeted delivery of “payloads” to the placenta while limiting maternal and fetal exposure. This review describes the current strategies, including placental targeting peptide-bound liposomes, nanoparticle or adenovirus constructs decorated with specific peptide sequences and placental gene promoters delivered via maternal IV injection, directly into the placenta or the uterine artery, as well as noninvasive site-selective targeting of regulating genes conjugated with microbubbles via contrast-enhanced ultrasound. The review also provides a perspective on the effectiveness of these technologies in various animal models and their practicability and potential use for targeted placental delivery of therapeutics and genes in adverse human pregnancies affected by placental dysfunction.

## 1. Introduction

The establishment of the placenta and appropriate uteroplacental blood flow are critical for fetal development, maternal well-being, and the physiologic homeostasis of offspring. During the first trimester of human and nonhuman primate pregnancy, placental extravillous trophoblast (EVT) migrate to and invade the maternal uterine spiral arteries. EVT replaces the vascular smooth muscle and endothelial cells, thereby transforming the spiral arteries/arterioles into high-capacity low resistance vessels to promote perfusion of the placenta [1,2,3,4,5]. Failure of spiral artery remodeling (SAR) results in placental ischemia and poor perfusion, a condition that underpins a number of pregnancy complications, including fetal growth restriction (FGR), new-onset maternal hypertension, early-onset preeclampsia, premature birth, and placental abruption [6,7,8]. Premature infants, offspring of preeclamptic pregnancies, and low birth weight babies have an increased risk for the development of cardiovascular disease, hypertension, type 2 diabetes, and adiposity [9,10,11,12,13,14,15,16,17,18,19]. In addition, mothers who develop preeclampsia often experience life-threatening complications, and worldwide maternal mortality consequent to preeclampsia approximates 50,000 each year [20,21,22]. Despite the high incidence of adverse pregnancies, current treatment options for preeclampsia or FGR are limited and, when indicated by fetal monitoring, induction of labor/early fetal delivery requires extensive neonatal intensive care and often leads to adverse health outcomes in adulthood. Maternal administration of drugs to treat hypertensive disorders leads to global distribution to multiple organs/tissues and often results in untoward side effects, as well as potential distribution to and effects on the fetus. Accordingly, the development and use of drugs to treat adverse conditions of pregnancy [23,24] are limited, and only a handful of drugs have been approved for use in pregnancy over the past 30 years [24,25].

Finally, although specific genetic modification of the rodent trophoblast lineage has been employed to gain a better understanding of the impact of genetic manipulation on placental development [26,27,28], applicability to the human remains unclear. Moreover, it is well-established that the placenta produces a number of physiologically active factors, e.g., vascular endothelial growth factor (VEGF), insulin-like growth factors; placental growth factor; placental lactogen, that regulates maternal vascular function and metabolism as well as the placental transfer of nutrients to the fetus [29]. Because these factors elicit both endocrine as well as paracrine effects, correction of a reduction in placental production and/or function of these factors has proved difficult to achieve. Systemic adenoviral gene or protein therapy in rodent and sheep models to increase directly/indirectly availability of growth factors, e.g., VEGF, can restore maternal vascular dysfunction, but the translation to the human and impact on fetus/offspring are unknown [28,30,31,32,33,34]. Accordingly, research direction has shifted to the development of gene therapy technologies [35] that provide targeted delivery of “payloads” to the placenta to treat the root cause of abnormal pregnancy while concomitantly limiting maternal and fetal exposure. This review will summarize the current strategies and their effectiveness in various animal models and discuss their practicability and potential use for targeted placental delivery of therapeutics, including genes in adverse human pregnancies affected by placental dysfunction.

## 2. Targeting Strategies

### 2.1. Peptide-Mediated Placental Targeting

Affinity-based peptide targeting was originally developed to achieve delivery of therapeutics to tumors and associated vasculature [23,36,37]. As discussed by King et al. [23], tumors express cell surface antigens typically absent from healthy tissues/vessels [37,38] that can bind circulating ligands, including peptides and antibodies [23,39]. Thus, systemic injection of such ligands to which a drug or gene is attached results in targeted delivery of the therapeutic to the tumor and not to normal cells. The placenta shares a number of features with solid tumors, e.g., the ability to undergo rapid cell proliferation, produce growth factors and cytokines, and evade immune surveillance [23,40]. Moreover, placental EVT, which migrate to and invade the uterine spiral arteries, behave like metastatic cancer cells. Therefore, King et al. [23] determined whether the tumor homing peptide sequences CGKRK or CRGDKGPDC affixed to liposomes bound to antigens specifically expressed on the rodent and human placental surface provide a means to deliver drugs/genes specifically to the placenta. Initial studies showed an injection of peptides labeled with carboxyfluorescein into pregnant mice were detected 3 h later attached to cells that comprise the placental labyrinth zone (site of nutrient exchange) and to the spiral arteries in the decidua (maternal placenta), but absent from other vascular beds and tissues except for the kidney from which they were excreted. Immunochemistry confirmed the attachment of peptides to the endothelium of non-remodeled spiral arteries and to the endovascular trophoblast lining remodeled arteries. CGKRK labeled peptide also accumulated in the syncytiotrophoblast, but not cytotrophoblast, of early gestation and term human placental explants. Additional studies showed that IV injection on days 11.5, 13.5, 15.5, and 17.5 of gestation of liposomes containing the peptide CRGDKGPDC to which IGF-2 was attached enhanced placental but not fetal weight in wild type animals and restored fetal weight in mice with FGR elicited by deletion of the U2 exon within the IGF-2 gene. Moreover, these effects of the IGF-2 peptide decorated liposomes were significantly greater than that elicited by the injection of IGF-2 alone or IGF-2 in empty (no peptide attached) liposomes. Finally, IGF-2 liposomes had no effect on mean litter size, the number of fetal resorptions, or the weight of maternal kidney or spleen, indicating minimal off-target accumulation of IGF-2 and that peptide-liposomes are well tolerated during pregnancy.

Micro RNAs (miRNAs) exert effects on the growth of the human placenta and fetus [41,42] and expression of miRNAs is altered in adverse pregnancies, including preeclampsia and FGR [43,44,45,46]. Therefore, using a similar peptide-based strategy, Beards et al. [24] determined whether targeted delivery of placental miR-145 or miR-675 inhibitor-conjugates could overcome the inhibitory effects of these respective miRNAs on human cytotrophoblast proliferation in vitro [47] and placental growth in mice [48]. Accordingly, liposomes decorated with the peptide CCGKRK to which was attached a synthetically produced miR-145 or miR-675 inhibitor peptide nucleic acid conjugate (PNA), were injected IV into mice on days 12.5, 14.5, and 16.5 of gestation or added to explants of the 1st trimester or term human villous placenta. Fetal and placental weight were either increased or normalized, i.e., decreased the variance and/or the number of fetal/placental weights below the 10th percentile, in pregnant mice injected with miR-145 inhibitor PNA or miR-675 inhibitor PNA compared to mice injected with scrambled control PNA. Neither control nor the miR-inhibitor PNA had any significant effect on litter size or the number of fetal resorptions. Incubation of human first trimester, but not term, placental explants with peptide targeted miR-145 or miR-675 inhibitor PNA significantly increased cytotrophoblast proliferation. The miR-675 inhibitor PNA also reduced placental miRNA 675, but not miRNA 145, expression in mice and placental explants.

**Figure 1:**
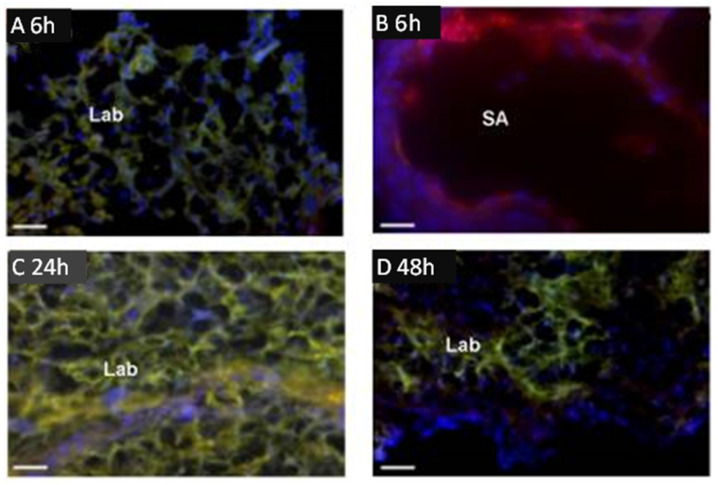
Binding of fluorescent dye-labeled CNKGLRNK to spiral arteries (SA) and labyrinth (Lab) of rodent placenta. Placentas from pregnant C57BL/6J mice collected at E18.5 following tail vein injection of Rh-CNKGLRNK (red)-decorated liposomes composed of NBD-labelled lipids (green) 6 (**A**,**B**), 24 (**C**), and 48 h (**D**) prior to tissue harvest. Yellow, co-localization of peptide (red) and lipid (green) fluorescence. Blue, DAPI (nuclei). Scale bars = 50 µM. Modified from [49].

Of particular interest is a study by Cureton et al. [49], who employed the placental specific surface peptide sequence NKGLRNK (NKG) to target the vasodilator 2-[[4-[(nitrox)methyl]benzoyl] thio]-benzoic acid methyl ester (SE175) to the placenta in mice in vivo and human placenta in vitro. Injection of fluorescent dye-labeled NKG or NKG to which cysteine was attached (CNKG) into pregnant mice resulted in localization almost exclusively to the intima of decidual spiral arteries and the vasculature of the mouse placental labyrinth zone (Figure 1). Importantly, peptides did not accumulate in the placental junctional zone, the vascular bed of any other maternal organ or in any fetal tissue.

Injection of liposomes decorated with CNKG to which the vasodilator SE175 was attached did not alter placental or fetal weight in wild-type mice. However, injection of peptide-targeted SE175 liposomes into eNOS^−/−^ mice, animals that exhibit pre-pregnancy hypertension, FGR, and narrow maternal spiral arteries, increased fetal weight and placental efficiency (i.e., fetal:placental weight ratio), eliminated oxidative stress and restored decidual spiral artery diameter to normal. None of these parameters was altered by injection of SE175 alone. CNGK-SE175 liposomes also induced vasodilation of mouse uterine arteries and human early gestation chorionic arteries in vitro and accumulated in the syncytiotrophoblast, but did not alter several parameters of syncytiotrophoblast function, e.g., transport of nutrients. As suggested by Cureton and colleagues [49], exploiting specific vascular targeting peptides to selectively deliver vasodilator to the uteroplacental vasculature may represent a novel treatment for FGR resulting from impaired uteroplacental perfusion.

Collectively, the studies outlined above clearly demonstrate the opportunity to exploit peptide-mediated targeting to deliver therapeutics to the placenta and utilize methodologies, e.g., phage screening, to identify peptide sequences specific to discrete cell populations. Future studies with the use of peptide-homing to deliver other therapeutics, e.g., DNA, mRNA, to restore gene-directed deficiencies in synthesis of key proteins important to placental development would prove useful. Indeed, Valero and colleagues [50,51] demonstrated that liposomal preparations, including the lipopthiourea DDSTU, which interacts with nucleic acids via hydrogen-bond interaction, and a pH switchable CSL-3 lipid that can complex silencing (si) RNA, are taken up by human placenta in vitro and release siRNA/nucleic acid. Clearly, as indicated by King et al. [23], peptide-directed targeting provides a novel platform for the development of placenta-specific therapeutics, including gene delivery.

### 2.2. Trophoblast Targeted Nanoparticles

As reviewed by Saunders [52], engineered nanoparticles (NP) have been used to access and deliver drugs/therapeutics, including DNA to specific tissue beds. In contrast to toxicologic/environmental NP which can be very harmful to humans, NP can be engineered to facilitate penetration of biological barriers and have biologically relevant sizes, e.g., DNA [53,54,55]. Accordingly, engineered NPs have been developed to treat various cancers [52,56,57]. Moreover, Semmler-Behnke et al. [58] showed that gold NPs accumulate in the rat placenta, but unfortunately also in the fetus, and engineered NP accumulate in vitro in transformed human placental cells. Recently, trophoblast targeted NP drug delivery has been achieved using engineered NP coated with a synthetic placental chondroitin sulfate A binding peptide (plCSA) or single-chain antibody fragments against the EGF receptor [59,60,61]. Thus, Zhang et al. [59,60] showed that plCSA NP bound specifically to trophoblast cells in human and mouse placenta in vitro, but not the placental junctional zone, decidua, fetus, or any maternal tissues, following IV injection to mice on days 6.5 to 14.5 of gestation. Moreover, plCSA NP containing methotrexate significantly reduced placental growth, induced placental apoptosis, and markedly compromised fetal development but had no effect on maternal tissues. In contrast, maternal tissues, notably the liver and kidney, were markedly impaired in animals treated with methotrexate alone. Collectively, these studies suggest that plCSA binding protein-guided NP could serve as a novel placenta-specific drug/gene delivery system.

**Figure 2:**
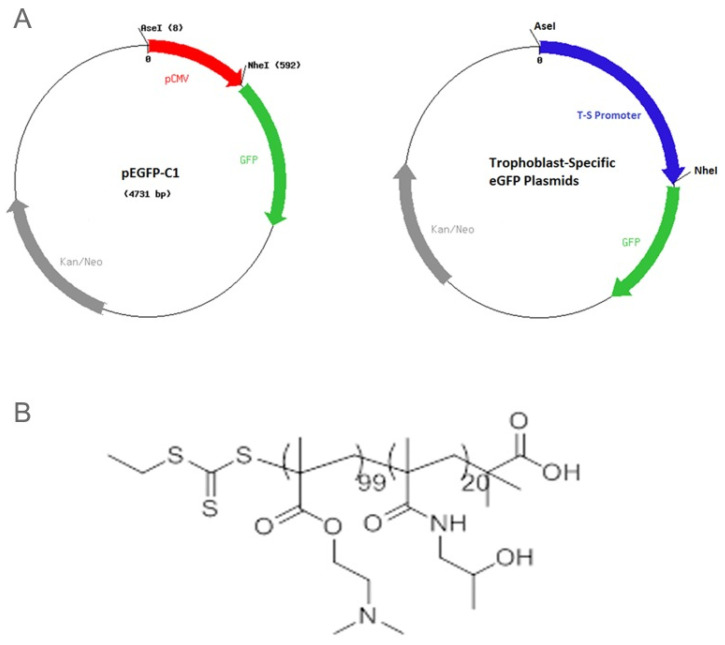
(**A**) Maps of the CMV-eGFP and trophoblast-specific plasmids and (**B**) the HPMA-DMAEMA copolymer used for DNA delivery in vitro and in vivo. Reproduced with permission from [62].

**Figure 3:**
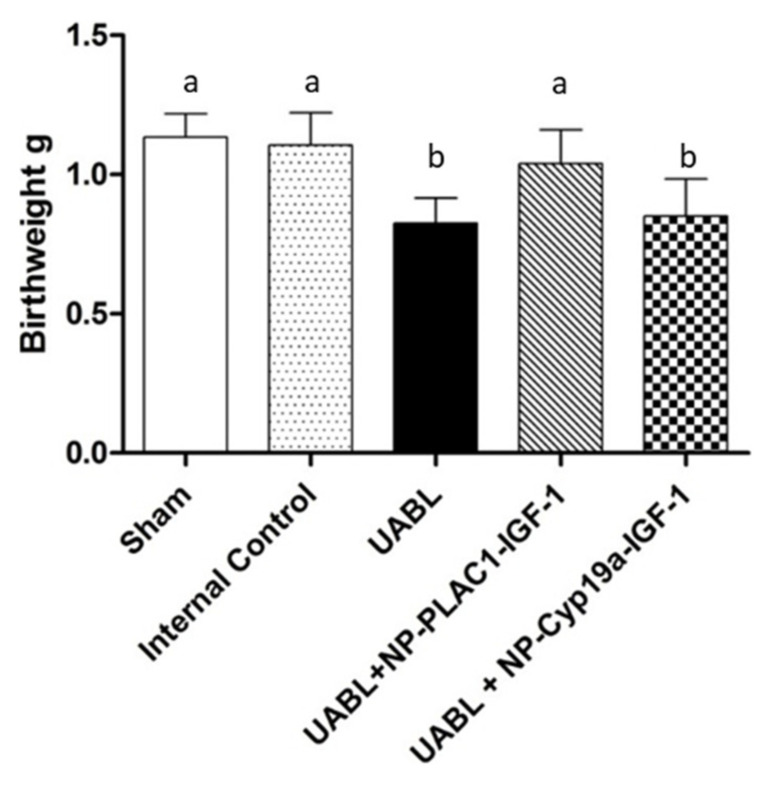
Offspring birthweights at delivery in sham-operated, internal control, and uterine artery branch ligated (UBAL) animals untreated or treated with PLAC1-HuIGF-1 nanoparticle or Cyp19a-HuIGF-1 nanoparticle. Modified from [62].

In an elegant study, Abd Ellah and colleagues [62] developed a nanostructure diblock delivery system complexed with the IGF-1 gene and specific placental gene promoters as a model for in utero gene therapy for FGR compromised by poor placental perfusion. Briefly, a non-viral nanoparticle comprised of poly [2-hyroxypropyl] methacrylamide (HPMA) and poly (2-(*N*,*N*-dimethylamino) ethyl methacrylate (DMAEMA), to which was complexed a plasmid DNA containing a specific sequence for IGF-1 as well as two fragments of the placental CyP19a promoter, the PLAC1 promoter or a CMV promoter, were prepared as outlined in Figure 2. HPMA is a water-soluble polymer that increases delivery of IGF1 through absorptive endocytosis and is biocompatible and non-immunogenic [53,54,55]. DMAEMA is a tertiary amine that acts as a weak base capable of being protonated at biological pH [54]. Initial in vitro studies showed the NP-GFP or NP-IGF-1 complexed with PLAC1 or Cyp19a increased the expression of GFP or IGF-1 mRNA in BeWo cells, but not in HEK293 kidney, uterine fibroblast or placental endothelial cells, confirming cell specificity of the NP targeted diblock preparation under control of the trophoblast specific promoters. In contrast, NP-GFP or NP-IGF-1 under control of a CMV promoter increased GFP/IGF-1 in all cell types transfected. Importantly, low birth weight induced in mice by uterine artery ligation was restored to normal following intra-placental injection of NP-PLAC1-IGF-1 (Figure 3). These experiments indicate that cell-specific gene expression using engineered NP-IGF-1 under control of placental cell-specific promoters was effective in restoring fetal growth in an in vivo mouse model of reduced placental perfusion. Moreover, Wilson and colleagues [63] showed that IGF-1 expression and key aspects of placental function were increased in human placental trophoblast cells and placental explants transfected with the PLAC1-IGF-1 NP. However, it remains to be determined whether placental targeting of IGF-1 can be achieved by peripheral administration of the NP diblock coated with tumor-homing peptides.

### 2.3. Adenovirus Mediated Intra-Placental Gene Therapy

**Figure 4:**
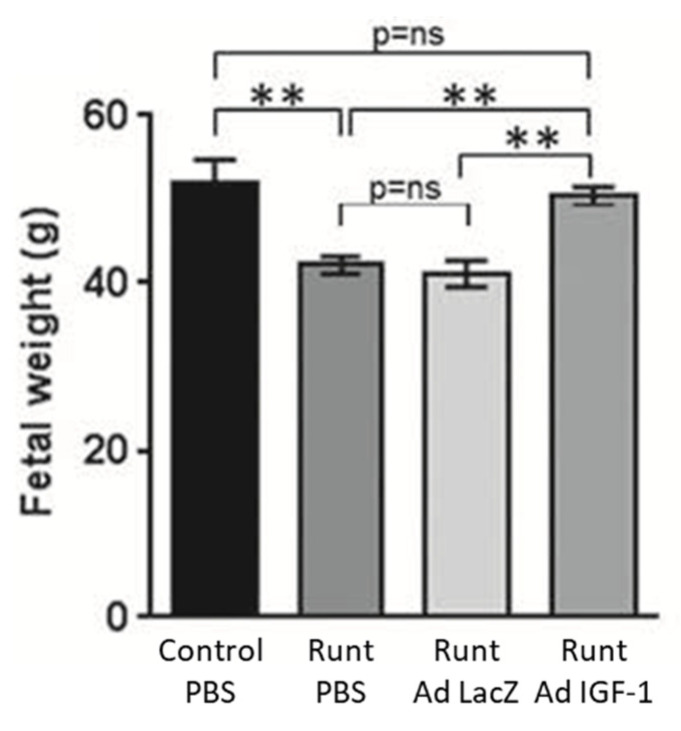
Effect of Ad-IGF-1 gene therapy in a rabbit model of IUGR in which pups at position 3 (Runt) were growth restricted. Ad-IGF-1 treatment significantly increased fetal weight of the runts to that of normally growing pups at the first position. ** *p* < 0.01. Modified from [70].

Adenovirus has received considerable attention to serve as an effective gene delivery vector because of its well-defined biology, genetic stability, and relatively high gene transduction efficiency since most human cells express the primary adenovirus receptor and secondary integrin receptors [64,65,66]. However, the latter complicates specificity in targeting and the need to develop modified viral capsids as well as circumvent anti-adenoviral vector immunity [66]. Accordingly, the impact of adenovirus-mediated delivery of the IGF-1 gene by intra placental injection on fetal growth was studied using in vivo rodent models and immortalized placental cell cultures [67,68,69,70]. Using a rabbit model of naturally occurring runt fetus at position 3, Keswani et al. [67,70] showed that intra-placental injection on day 21 of gestation of an E1-deleted adenoviral construct encoding the human IGF-1 gene driven by a CMV promoter restored fetal body, liver, and musculoskeletal weight, but had no effect on the weight of the normal first position fetus or the placenta (Figure 4). Additional studies confirmed IGF-1 protein expression after gene transfer along with the maternal-placental interface but minimal gene transfer to fetuses or maternal organs.

Intraplacental delivery of a similar adenoviral human IGF-1-CMV promoter constructs into mice in which placental insufficiency and FGR were created by uterine artery branch ligation resulted in increased levels of IGF-1 in placentas and restoration of fetal weight [68,69] and prevented the development of FGR-induced cardiac dysfunction in offspring [71]. Additional studies showed the effects of placental IGF-1 over-expression appeared to reflect regulation of placental expression and/or localization of glucose transporters [69] as well as key amino acid transporters [68]. Similar but not congruous effects were detected in BeWo cells transfected with the adenoviral human IGF-1-CMV construct [68,69].

### 2.4. Adenovirus Mediated Maternal Intrauterine Arterial Gene Therapy

The effects of adenovirus-mediated vascular endothelial growth factor (VEGF-A165) gene therapy delivered via the maternal uterine artery was examined in a sheep model of FGR induced by high caloric intake initiated early in gestation [72]. Accordingly, at approximately 60% of gestation, high calorie intake ewes received bilateral uterine arterial intravascular injection of replication deficient (E1, E3 deleted) adenovirus containing the VEGF-A165 gene (Ad.VEGF) or the β-galactosidase gene (Ad.LacZ) or saline. Normal caloric fed pregnant sheep received a saline vehicle. Uterine arterial Ad.VEGF, but not saline or Ad.LacZ, treatment increased fetal growth velocity as assessed by ultrasound within 3–4 weeks, and fewer fetuses were growth restricted at 90% of gestation. Moreover, the fetal to placental weight ratio, an index of placental efficiency, as well as mRNA levels of VEGF receptors 1 (FLT1) and 2 (KDR) in the maternal but not the placental compartment, were significantly greater in Ad.VEGF pregnancies. Ultrasound also indicated that fetal biparietal to abdominal circumference and brain to liver weight ratios were lower in Ad.VEGF injected animals suggesting fetal brain sparing. In otherwise normal pregnant sheep, injection of Ad.VEGF to the uterine artery has previously been shown to increase uterine blood flow [73,74]. Interestingly, however, in the study of high caloric-induced FGR, uterine blood flow was not altered by Ad.VEGF, although 2nd and 3rd order uterine arterial vessels exhibited a greater degree of relaxation in vitro. The authors suggested that their study provides first proof of principle of Ad.VEGF therapy via maternal uterine arterial injection to treat FGR, although the exact mechanism remains to be determined [72].

**Figure 5:**
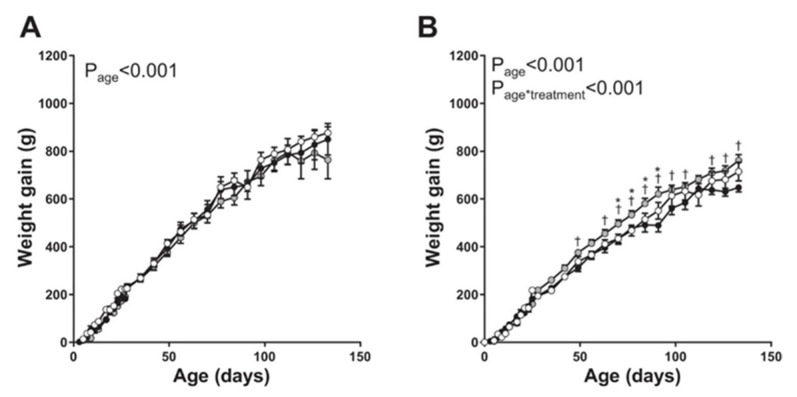
Postnatal weight gain in male (**A**) and female (**B**) offspring of sham-treated control sows (open circles; *n* = 7 male pups, *n* = 13 female pups), offspring of sows undernourished to induce FGR (solid circles; *n* = 6 male pups, *n* = 13 female pups), and offspring of sows undernourished and given adenoviral vascular endothelial growth factor A_165_ (Ad.VEGF-A_165_) gene therapy (shaded circles; *n* = 8 male pups, *n* = 10 female pups). * *p* < 0.05 vs. controls; † *p* < 0.05 vs. untreated FGR at same age. Modified from [75].

Vaughan et al. [75] determined the perinatal as well as long-term effects on offspring vascular function elicited by maternal uterine artery adenoviral VEGF-A 165 gene therapy at mid-gestation in a maternal caloric restricted model of FGR in the guinea pig. However, in this study, the Ad.VEGF construct was applied to the surface of the uterine artery (i.e., extravascular) and not injected into (intravascular) the artery. Compared with the decrease in fetal weight in untreated caloric restricted pups, gene therapy-induced a small but significant increase in birth weight of male and female pups. Moreover, although there were no major impacts of caloric restriction or Ad.VEGF gene treatment on blood pressure, adrenal weight, or basal and stimulated adrenal cortisol production in male and female offspring, postnatal weight gain was 10–20% greater in female offspring of caloric restricted Ad.VEGF-treated animals (Figure 5). Thus, as concluded by the authors, increased fetal growth conferred by maternal artery Ad.VEGF therapy was sustained in female offspring.

Collectively, the results of these studies and other complementary studies on the impact of VEGF gene uterine arterial injection are encouraging and potentially applicable to human FGR in general and severe FGR in particular. However, the invasive uterine arterial injection was employed and none of the studies examined mechanisms or treated the apparent root cause, namely poor placental perfusion, thought to underpin the ontogenesis of FGR. Nevertheless, since uterine arterial Ad.VEGF treatment appeared effective, a multinational, multidisciplinary collaboration to carry out a phase I/IIa clinical trial that aims to examine the safety and efficacy of maternal gene therapy as a treatment for severe early onset FGR has been put forth and known as the EVERREST Project (does vascular endothelial growth factor gene therapy safely improve outcome in severe early-onset fetal growth restriction) [76,77].

### 2.5. Targeted Placental VEGF Gene Therapy to Restore Uterine Artery Remodeling (UAR) in a Nonhuman Primate Model of Defective UAR

**Figure 6:**
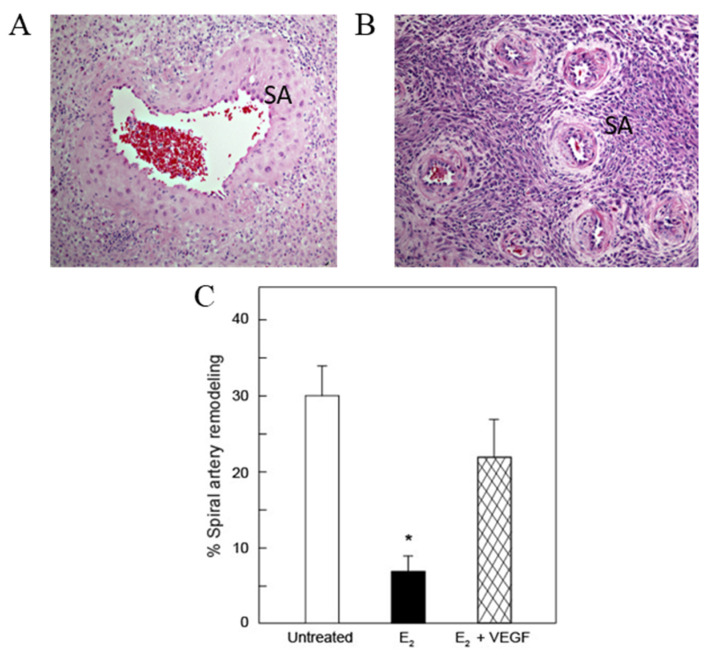
Photomicrographs of H & E histology of the basal plate and spiral arteries (SA) in untreated (**A**) and estradiol-treated (**B**) baboons. (**C**) Mean (± SE) percentage of uterine spiral arteries remodeled (i.e., number of trophoblast invaded arteries divided by total number of arteries counted), as quantified by image analysis, for vessels greater than 50 µM in diameter on day 60 of gestation in untreated (*n* = 13), estradiol (E_2_)—treated (*n* = 15) and E_2_ plus VEGF gene-treated (*n* = 6) baboons. * *p* < 0.001 versus untreated and E_2_ plus VEGF gene treated animals. Modified from [82].

Using the baboon as a translational model, our laboratories have shown that the low level of ovarian estradiol (E_2_) during the first trimester of nonhuman primate pregnancy is essential for promoting UAR [78,79]. Thus, simply shifting the normal rise in E_2_ from the second to the first third of pregnancy suppressed UAR. Figure 6 illustrates the marked architectural EVT remodeling of a spiral artery into distensible low-resistance vessels with enlarged lumens in an untreated baboon (A) and the highly-coiled high-resistance non-remodeled spiral arteries of an estradiol-treated baboon (B) on day 60 (term = day 184). Image analysis confirmed that the percentage of uterine spiral arteries remodeled by cytokeratin-positive EVT (Figure 6) was 4-fold lower on day 60 in estradiol-treated (7 ± 2%) than in untreated (30 ± 4%) animals [78,79]. The impairment of UAR induced by E_2_ treatment was also associated with a significant decrease in placental EVT expression of VEGF [78,79]. Moreover, UAR-impaired baboons exhibit the complications of early-onset preeclampsia, including maternal vascular endothelial dysfunction, decreased capillary density, luminal area and eNOS expression in systemic skeletal muscle microvessels, impaired flow-mediated brachial artery vasodilation and hypertension [80]. In more recent studies, we have also utilized a highly innovative real-time imaging technology, B-Flow/Spatio-temporal image correlation M-mode, to noninvasively assess spiral artery luminal diameters at systole and diastole as a measure of the extent of UAR/vessel distensibility in our baboon model [81]. Therefore, the early E_2_-treated baboon provides a novel primate experimental model to study the impact of impaired UAR on maternal and fetal well-being and development and physiologic function of offspring. Moreover, having a model of UAR permits study of the potential of placental gene therapy to correct UAR and resultant clinical pathologies.

Contrast-enhanced ultrasound (CEU) imaging and mediated cavitation of acoustically active microbubble (MB) carriers (CEU/MB) has been employed in cardiac diagnostic medicine and to quantify placental microvascular perfusion in humans and macaques [83,84]. CEU/MB has also emerged as an innovative strategy to visualize and target deliver genes in vivo to specific tissues [85,86,87,88,89]. Therefore, we used our nonhuman primate model of prematurely elevating E_2_ and CEU/MB to deliver VEGF DNA specifically to the placental basal plate/maternal aspect of the placenta during early baboon pregnancy to establish the role of VEGF in regulating UAR [82].

**Figure 7:**
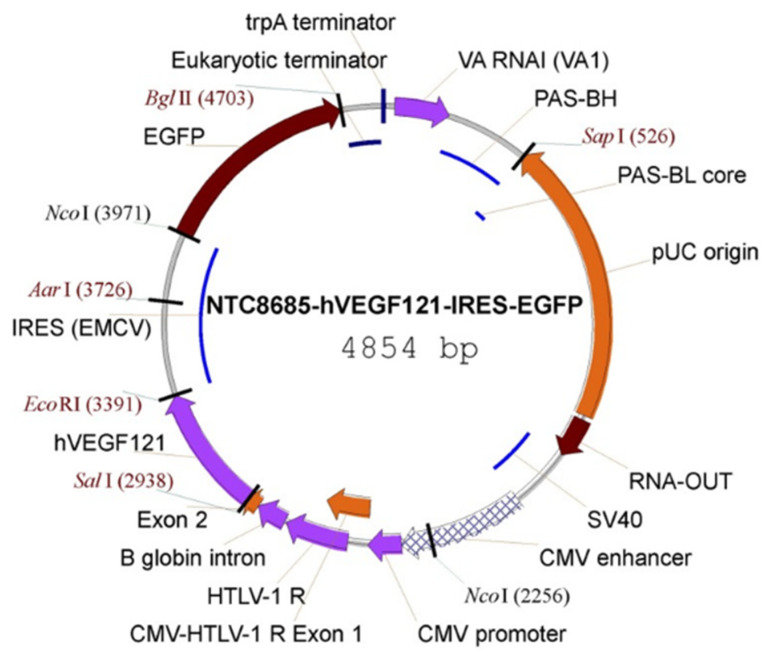
Vector map of the NTC8685–human (h)VEGF_121_–IRES–EGFP. CMV, cytomegalovirus; EGFP, enhanced GFP; HTLV-1, human T-cell leukemia virus type 1; IRES (ECMV), internal ribosomal entry site (from the encephalomyocarditis virus); PAS, primosomal assembly site; PAS-BH, primosomal assembly site sequence on the pBR322 H strand; PAS-BL, primosomal assembly site sequence on the pBR322 L strand; pUC, plasmid University of California; SV, simian virus; trpA, tryptophan A; VA RNAI, virus-associated RNA I, a type of noncoding RNA that plays a role in regulating translation. Reproduced with permission from [82].

**Figure 8:**
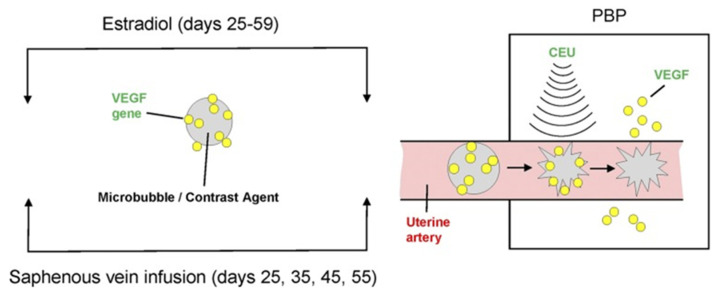
Illustration of CEU/MB-targeted delivery of the VEGF gene to the placental basal plate (PBP) of E_2_-treated baboons during early pregnancy. Reproduced with permission from [82].

**Figure 9:**
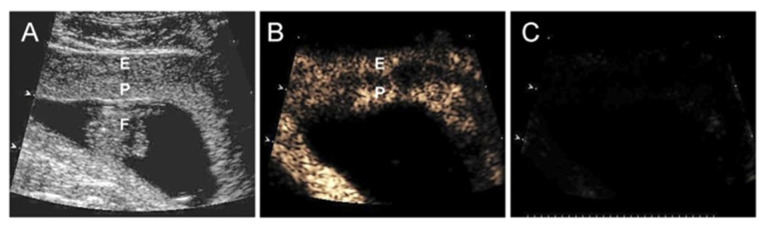
(**A**) Grayscale Doppler image of the endometrium (E), placenta (P), and fetus (F), and CEU images (**B**) before and (**C**) after the collapse of the MBs/uncoupling of VEGF DNA on day 45 of gestation in an E_2_-treated baboon. Reproduced with permission from [82].

Cationic lipid-encapsulated/decafluorobutane-filled MBs (~2 µM diameter) were charge conjugated with a bicistronic plasmid vector encoding VEGF_121_ (Figure 7) and GFP and infused into a maternal saphenous vein of estradiol-treated baboons for 10 min on days 25, 35, 45, and 55 of gestation (Figure 8). A 2.0–6.0 MHz 6 C2 transducer and resting mechanical index (negative acoustic pressure) of 0.2 was positioned on the abdomen during infusion, and the ultrasound beam was directed to the placental basal plate, identified by 2D imaging. The mechanical index was then increased to 1.9 with repeated 5 s burst pulses during the 10 min delivery period to collapse the MBs and thus detach/release the VEGF DNA within the placental basal plate [82]. Figure 9 shows a grayscale Doppler image of the endometrium, placenta, and fetus (panel A) and CEU images showing the presence of VEGF DNA-labeled MBs/contrast agent in the endometrium and placental basal plate but not the fetus before (panel B) and absence at the end (panel C) of a 5 s burst and collapse of the MB to release VEGF DNA. As seen in Figure 6, UAR on day 60 was largely restored by VEGF gene delivery (22 ± 5%), establishing for the first time the important role of VEGF in regulating UAR in vivo during primate pregnancy and the ability to target the VEGF gene to the placental basal plate in early pregnancy to correct impaired UAR [82].

An important feature of these studies is the ability to selectively deliver the VEGF gene to a specific region of the maternal aspect of the placenta in a noninvasive manner and repeatedly during gestation [82]. Treatment did not impair fetal growth nor induce any maternal problems, e.g., premature birth. Moreover, the VEGF gene did not cross the placental barrier and thus was not detected in the fetus. Most importantly, the animal model [90] provides a novel approach to directly study the impact of UAR per se and therapeutic gene delivery in the prevention of impaired UAR and the pathologies associated with poor placental perfusion in a nonhuman primate with translational impact to human pregnancy.

## 3. Summary

It is apparent that significant progress has been made in the development of liposomal, engineered nanoparticle and adenovirus constructs in which sequences for various genes, e.g., IGF1, VEGF as well as miRNA inhibitors, growth-promoting peptides, and drugs to control vascular tone, have been inserted. Moreover, studies have shown that intraplacental and/or intrauterine arterial injection of constructs containing IGF-1 or VEGF gene sequences driven by specific placental promoters increased placental synthesis of regulatory gene product protein and restored fetal and/or placental dysfunction in rodent and sheep models in which the clinical manifestations of poor placental perfusion, e.g., FGR, were elicited by maternal caloric modification or uterine arterial ligation. Moreover, it would appear that these and other constructs containing growth-promoting peptides or miRNAs are retained in the placenta and not transferred to the fetus or the maternal circulation. However, target specificity was achieved by injection of constructs into the placenta or the uterine artery. In this context, the development of affinity-based peptide targeting constructs, e.g., placenta-specific peptide sequences affixed to liposomes/nanoparticles, is most interesting and the methodology provides a novel platform for the development of placenta-specific therapeutics. Indeed, maternal IV injected liposomes decorated with tumor homing or placenta-specific peptide sequences were sequestered in the labyrinth zone of the rodent placenta and spiral arteries and absent from any maternal vascular beds. Moreover, constructs containing IGF-2 protein restored fetal growth in IGF-2 knock-out mice, while those containing a potent vasodilator restored vascular tone and spiral artery diameter in eNOS deficient mice.

Collectively, the latter studies are extremely powerful and have substantially enhanced the field of targeted placental therapy for the complications of adverse pregnancies. However, although complementary in vitro studies have shown that constructs are functional in primary or immortalized human placental cells or explants, it remains to be determined whether results in rodents and sheep are translatable to humans considering the marked differences in placentation and fetal-placental-maternal interaction between these species and humans/nonhuman primates. Moreover, the studies cited did not examine mechanisms or treat the root cause of several cases of adverse pregnancy, namely poor placental perfusion due to deficient spiral artery remodeling. Nonhuman primate models in which UAR can be experimentally suppressed and prevented by noninvasive targeted delivery of regulatory genes permit the study of the impact of UAR per se, respective underlying mechanisms, therapeutic value of gene delivery, and translational impact to human pregnancy.

## Data Availability

Not applicable for this review.
